# It's Probably Mine: Self‐Prioritization Can Be a Decisional Strategy

**DOI:** 10.1111/cogs.70190

**Published:** 2026-03-26

**Authors:** Marius Golubickis, Esther S. Selvaraj, Siobhan Caughey, Parnian Jalalian, Yadvi Sharma, C. Neil Macrae

**Affiliations:** ^1^ Department of Cognitive Sciences United Arab Emirates University; ^2^ School of Psychology University of Aberdeen; ^3^ Alliance Manchester Business School University of Manchester

**Keywords:** Self, Ownership, Self‐prioritization, Egocentrism, Heuristics, Decision‐making, Drift diffusion model

## Abstract

Personal possession exerts a significant influence on decision‐making, such that stimulus classification is speeded when objects belong to the self (vs. other persons). Exactly when and how this self‐prioritization effect arises, however, remains a matter of speculation and debate. Accordingly, adopting a psychophysical approach in combination with computational modeling, here we hypothesized that self‐prioritization could derive from the application of an egocentric strategy (i.e., default‐to‐self response) during decisional processing. Using a modified object‐classification task in which participants judged blended images comprising varying amounts of self‐owned and friend‐owned objects (i.e., pencils and pens), the results of two experiments supported this viewpoint. Participants perceptually prioritized their possessions only when a self‐centric decisional strategy was applicable (Experiment 1), an effect that was amplified when the demands of decision‐making increased (Experiment 2). Additional computational analyses traced the origin of self‐prioritization to a prestimulus preference for self‐related responses, a strategic component of decision‐making. Collectively, these findings inform understanding of how ownership influences decisional processing, with wider implications for theoretical accounts of self‐bias.

A basic facet of mental life is that self‐relevance facilitates information‐processing and response generation (Humphreys & Sui, 2016; Sui & Humphreys, 2015, 2017). Take, for example, the influence that personal possession exerts on decision‐making (Morewedge, 2021). As a variant of the aptly termed self‐prioritization effect (SPE), stimulus classification is speeded when objects belong to the self (vs. targets of comparison), a benefit that emerges even when the items in question are inconsequential (Falbén et al., 2019; Falbén et al., 2020; Golubickis, Falbén, Cunningham, & Macrae, 2018, 2019, 2021, 2023). Notwithstanding repeated demonstrations of this SPE, outstanding issues nevertheless remain. Prominent among these are questions concerning how exactly personal possession impacts decisional processing and through which theoretical lens SPEs of this kind can be characterized. Addressing these matters, here we adopted a psychophysical approach in combination with computational modeling to provide a comprehensive process‐based account of when and how ownership influences decision‐making.

## 1 Routeways to self‐prioritization

In standard object‐ownership tasks, participants are presented with trivial items (e.g., stationery, mugs) that purportedly belong to self and a friend (or mother/stranger) with the goal of classifying the stimuli (e.g., owned‐by‐self vs. owned‐by‐friend) as quickly and accurately as possible (Golubickis et al., 2018). A reliable finding has emerged in this work. Objects owned by self are classified more rapidly than comparable items belonging to a friend (i.e., self‐prioritization), a direct demonstration of the advantage that personal possession exerts on decision‐making (Falbén et al., 2019; Falbén et al., 2020; Golubickis et al., 2018, 2019, 2021, 2023). Interestingly, this facilitatory effect parallels a more basic manifestation of self‐prioritization that has been observed in shape‐label matching tasks (Humphreys & Sui, 2016; Sui & Humphreys, 2015, 2017). Following the association between various person labels and geometric shapes (e.g., self = circle, friend = square, stranger = triangle), subsequent shape‐label classification judgments are faster for self‐relevant compared to other‐relevant stimulus pairings (Sui et al., 2012, 2013). Thus, when trivial objects are owned‐by‐self (vs. others) or geometric shapes serve as proxies for self (vs. others), decision‐making is enhanced (Golubickis & Macrae, 2023; Humphreys & Sui, 2016; Sui & Humphreys, 2015, 2017).

To identify the processes underpinning self‐prioritization, hierarchical drift diffusion modeling has been a popular analytical tool (Wiecki, Sofer, & Frank, 2013). Using both response latency and accuracy to represent the decision‐making process as it unfolds over time, the drift diffusion model (DDM) is useful as it identifies the latent cognitive operations that drive task performance (Ratcliff, Smith, Brown, & McKoon, 2016; Voss, Rothermund, Gast, & Wentura, 2013, 2015). During binary decision‐making (e.g., is an object owned‐by‐self or owned‐by‐friend?), information is continuously sampled from a stimulus until sufficient evidence has been accumulated to generate a response. The utility of the DDM lies in its ability to disentangle biases in stimulus and response‐related processes as decisions are made. Critically, these biases are conceptually distinct with different underlying origins and interpretations (Leite & Ratcliff, 2011; Mulder, Wagenmakers, Ratcliff, Boekel, & Forstmann, 2012; Ratcliff et al., 2016; van Ravenzwaaij, Mulder, Tuerlinckx, & Wagenmakers, 2012; Voss et al., 2013, 2015; White & Poldrack, 2014).

In terms of the DDM parameters that index bias, drift rate (*v*) estimates the speed of information uptake, thus is interpreted as a measure of the quality of stimulus processing during decision‐making (i.e., stimulus bias). Independently, boundary separation (*a*) estimates the distance between the two response thresholds (i.e., evidential requirements of response selection), and the starting point (*z*) specifies the position between the thresholds at which evidence gathering begins. If *z* is not centered between the thresholds, this denotes a bias in favor of the response that is closer to the starting point (i.e., response‐selection bias). Of theoretical significance, these stimulus and response‐related biases have different operational characteristics, hence implications for the interpretation of SPEs (Golubickis & Macrae, 2023; Humphreys & Sui, 2016). Whereas drift rate is driven by stimulus‐related properties and is not under subjective control, boundary separation and starting point are determined by the decision‐maker, and thus are controllable (Wagenmakers, 2009). What this, therefore, indicates is that self‐prioritization may be grounded in differences in the uncontrollable and/or strategic aspects of decision‐making.

Inspection of the extant literature indicates that both stimulus and response‐related biases have the capacity to generate self‐prioritization, what matters is how the effects of self‐relevance are probed across different decision‐making settings (Golubickis & Macrae, 2023; Humphreys & Sui, 2016; Sui & Humphreys, 2015, 2017). Based on a body of evidence garnered from shape‐label matching tasks, Sui and colleagues have argued that stimulus prioritization is supported by specialized attentional operations that enhance the perceptual salience of self‐relevant material, which in turn facilitates decision‐making (i.e., Self‐Attention Network [SAN], see Humphreys & Sui, 2016; Sui & Humphreys, 2015, 2017; Sui, Rotshtein, & Humphreys, 2013, 2023). Thus, according to this account, self‐prioritization reflects the operation of a stimulus bias (i.e., differences in drift rate), a viewpoint that has garnered considerable empirical support. When the task at hand is to judge whether shape‐label stimulus pairs correspond (i.e., match vs. mismatch) with previously learned associations, self‐prioritization is underpinned by speeded information uptake during decisional processing (Golubickis et al., 2017, 2020; Hu, Lan, Macrae, & Sui, 2020; Sui, He, Golubickis, Svensson, & Macrae, 2023; Svensson et al., 2022).

Interestingly, when the to‐be‐required judgment shifts from shape‐label correspondence to object ownership (i.e., self‐owned vs. friend‐owned), self‐prioritization has a different cognitive origin. Aside from a single exception (Falbén et al., 2020), work has revealed that self‐prioritization is underpinned by differences in the starting point of evidence accumulation (i.e., a prior bias toward self‐relevant responses), indicating that ownership influences decisional processing via a response‐related pathway (Falbén et al., 2020; Golubickis et al., 2018, 2019, 2021, 2023). Whether this reflects the strategic character of object‐ownership effects, however, remains uncertain as several issues warrant consideration. First, research to date has failed to employ decision‐making environments optimized to probe the tactical basis of self‐prioritization. To do so, experiments must create task settings in which a self‐centric decisional strategy varies in applicability (i.e., relevant vs. irrelevant), with the resulting data submitted to computational analyses to identify the critical underlying operations. Thus far, these issues have been explored independently (Caughey et al., 2021; Constable, Welsh, Huffman, & Pratt, 2019; Falbén et al., 2019; Golubickis et al., 2018). Second, contrasting work using shape‐label matching tasks in which self‐prioritization is grounded in the workings of the SAN model (Humphreys & Sui, 2016; Sui & Humphreys, 2015, 2017), little attempt has been made to link ownership‐based effects with established theoretical accounts of decision‐making (Morelli, Casagrande, & Forte, 2022; Santos & Rosati, 2015).

## 2 Self‐prioritization as a decisional strategy

A clue to how ownership potentially elicits self‐prioritization can be found in decades of social psychological research demonstrating that self commonly functions as an anchor or reference point during decision‐making. When motivation is low, attention is limited, or uncertainty is high, self‐centric responding (i.e., default‐to‐self response) is a tactic that simplifies decisional processing (Epley, Keysar, Van Boven, & Gilovich, 2004; Epley & Gilovich, 2004; Royzman, Cassidy, & Baron, 2003; Surtees & Apperly, 2012; Todd & Tamir, 2024). Just such a strategy, we suspect, may also be operating during object‐ownership tasks, at least under certain conditions. Simplifying decisional strategies are commonly deployed when they are appropriate and useful to the decision‐maker. According to the theoretical position advanced by Gigerenzer and colleagues, the mind is an adaptive toolbox comprising a repertoire of (fast and frugal) heuristics that are utilized when the benefits are greatest (Gigerenzer, 2008; Gigerenzer, Hertwig, & Pachur, 2011; Gigerenzer & Gaissmaier, 2011; Gigerenzer & Goldstein, 1996). Rather than optimizing decision‐making, people typically satisfice by selecting the solution that works best for them in the current task context, whatever that solution may be (Simon, 1955).

Derived from the mathematical modeling of decisional processing, fast and frugal heuristics are ecologically rational as they consider the computational capacities of the decision‐maker alongside the structural characteristics of the environment in which decision‐making takes place (cf. Tversky & Kahneman, 1974). Significantly, these heuristics are also computationally specifiable, providing a formal account of decisional processing. As an evolved capacity or acquired through experience, heuristics enable problems to be solved quickly and easily with minimal information. During object‐ownership tasks, self‐prioritization may emerge via the operation of such a decisional strategy—the “it's probably mine” heuristic. Specifically, prior to stimulus presentation, participants assume that a forthcoming object is more likely to belong to self than a target of comparison (e.g., friend, stranger), a presumption (i.e., default‐to‐self) that favors the selection of self‐related responses (White & Poldrack, 2014). Crucially, however, this only happens when the tactic is applicable (i.e., task‐relevant) given the problem confronting the decision‐maker (Constable et al., 2019; Falbén et al., 2019), with the strength of self‐prioritization determined by the demands of the environment in which decisional processing takes place (Gigerenzer & Gaissmaier, 2011).

Lending support to this viewpoint is the observation that egocentrism and personal possession are inexorably intertwined (Morewedge, 2021). Early childhood is characterized by an emerging understanding of ownership together with self‐centric patterns of reasoning and responding (Fasig, 2000; Hay, 2006). To function effectively in a complex social world, children must be able to parse external stimuli into items that are relevant to self, and items that are not. Although the misplaced belief that all objects are personally pertinent diminishes markedly with age, childhood egocentrism is never fully eliminated (Birch & Bloom, 2004; Nickerson, 1999; Royzman et al., 2003; Todd & Tamir, 2024). Indeed, among adults, self‐centric decisional strategies prevail in a variety of decision‐making settings (Epley et al., 2004; Gilovitch et al., 1998, 2000; Zhao, Zhang, Tao, Duan, & Xu, 2023). By extension, adoption of such a deeply rooted social‐cognitive stratagem would provide an effective means through which object‐ownership tasks could be performed, with self‐prioritization the resulting outcome. When operating, the computational signature of this decisional tactic would be a prestimulus preference for self‐owned (vs. friend‐owned) responses (Golubickis et al., 2018).

## 3 The current research

In two experiments, the current research explored the origins of self‐prioritization during an object‐ownership task. Following previous work (Golubickis et al., 2018, 2019), participants were presented with objects (i.e., pencils and pens) that allegedly belonged either to self or best friend, with the task of classifying the items based on ownership (i.e., self‐owned vs. friend‐owned). To conduct a fine‐grained examination of decisional processing, the to‐be‐judged stimuli comprised morphed images that varied on a continuum from 100% pencil to 100% pen (i.e., varying amounts of the self‐owned and friend‐owned objects were included in the images). Crucially, this psychophysical approach permitted the point of subjective equality (PSE) to be calculated for each participant—that is, the point at which owned‐by‐self and owned‐by‐friend responses were equally likely to be generated (i.e., the stimuli were perceived to be equivalent). Critical for the establishment of bias is the difference between the PSE and the point of objective equality (POE), which represents the actual physical value where stimuli are equal. If the PSE and POE differ, this indicates perceptual bias.

As the use of simplifying strategies is context dependent (Gigerenzer, 2008; Gigerenzer & Gaissmaier, 2011), in Experiment 1, the applicability of a self‐centric decisional tactic was manipulated by varying the object‐related judgment participants were required to make (Constable et al., 2019; Falbén et al., 2019). Whereas some participants judged to whom the items belonged (i.e., self or friend), others reported the identity of the objects (i.e., pencil or pen). Utilized when applicable, an egocentric decisional strategy (i.e., it is probably mine) was expected to be deployed only when judgments of ownership (vs. identity) were requested (i.e., people do not have an established preference for responses pertaining to items of stationery). In turn, the two judgment conditions were anticipated to yield different psychophysical response profiles. When judgments of ownership were provided—revealing self‐prioritization—the PSE was expected to occur when less self‐related compared to friend‐related information was included in the morphed image (i.e., the PSE was shifted from the POE). In contrast, when judgments of identity were furnished, no shift in the PSE was predicted. To identify the operations underpinning decision‐making, data were submitted to a hierarchical drift diffusion model (HDDM) analysis. Reflecting its strategic origin, self‐prioritization was expected to be grounded in a prestimulus preference for self‐owned (vs. friend‐owned) responses.

## 4 Experiment 1

### 4.1 Method

#### 4.1.1 Participants and design

Eighty participants (53 female, *M*_age_ = 21.67, *SD* = 2.73) took part in the research, all of whom had normal or corrected‐to‐normal visual acuity and received £10 (∼$13) for their assistance. Informed consent was obtained from participants prior to the commencement of the experiment, and the protocol was reviewed and approved by the Ethics Committee at the School of Psychology, University of Aberdeen, Scotland. The experiment had 2 (Judgment: ownership or identity) X 2 (Owner: self or friend) mixed design with repeated measures on the second factor. To detect a significant Judgment X Owner interaction with a medium‐to‐large (*d* = 0.65) effect size (Caughey et al., 2021; Constable et al., 2019), a minimum sample of 26 participants per Judgment condition afforded 80% power (PANGEA v0.2).

#### 4.1.2 Stimulus materials and procedure

Participants arrived at the laboratory individually, were greeted by a researcher, seated at a desktop computer, and informed that the experiment comprised an object‐classification task with two categories of stimuli: pencils and pens (Golubickis et al., 2018). Prior to the task, the computer assigned one category of object to the participant (i.e., owned‐by‐self) and the other category to their best friend (i.e., owned‐by‐friend). Thus, for the duration of the experiment, every pencil (or pen) was self‐owned, and every pen (or pencil) was friend‐owned. Object‐owner assignments were counterbalanced across the sample. Following the distribution of objects to self and friend, participants were randomly allocated to one of the judgment conditions. Whereas in the ownership‐condition, participants reported whether the depicted item belonged to self or friend, in the identity‐condition, they decided whether the item was a pencil or pen (Falbén et al., 2019). Judgment was manipulated between participants to avoid carryover and contamination effects that would arise if individuals performed both ownership and identity tasks in the same session. Participants were instructed to respond as quickly as possible using two keys on the keyboard (C and M), with key‐response mappings counterbalanced across the sample.

**Fig. 1:**
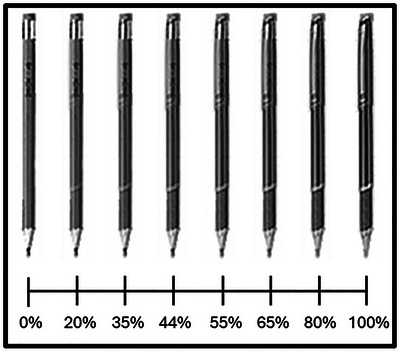
Example of a pencil‐to‐pen continuum. Each image depicts the same object morphed from a pencil (0% pen information) to a pen (100% pen information) in eight steps (0%, 20%, 35%, 45%, 55%, 65%, 80%, 100%).

Each trial began with the presentation of a central fixation cross for 1000 ms, followed by a to‐be‐judged object for 400 ms. After each object was presented, the screen turned blank until participants reported the owner or identity of the item. Following each response, after 500 ms, the fixation cross reappeared, and the next trial commenced. The to‐be‐judged stimuli comprised grayscale images of 20 writing implements, 10 pencils and 10 pens. Each image subtended 21 × 206 pixels, and the stimuli were equated for luminance. To create a continuum from pencil‐to‐pen, every photograph of a pencil was linearly blended with a photograph of a pen using Adobe Photoshop. Eight morph levels were generated for each stimulus pair by systematically replacing increasing proportions of pencil pixels with the corresponding pen pixels (0%, 20%, 35%, 45%, 55%, 65%, 80%, 100%), such that morph values reflected how much pen was represented in the image. Two unaltered photographs (0% and 100%) served as category anchors, yielding an eight‐step stimulus continuum for all 20 pairings (see Fig. 1). Participants initially performed 16 practice trials, followed by seven blocks of 80 trials in which all stimuli appeared equally often (i.e., 10 times per block). On completion of the task, participants were debriefed, thanked, and dismissed.

## 5 Results and discussion

### 5.1 Object classification

**Fig. 2:**
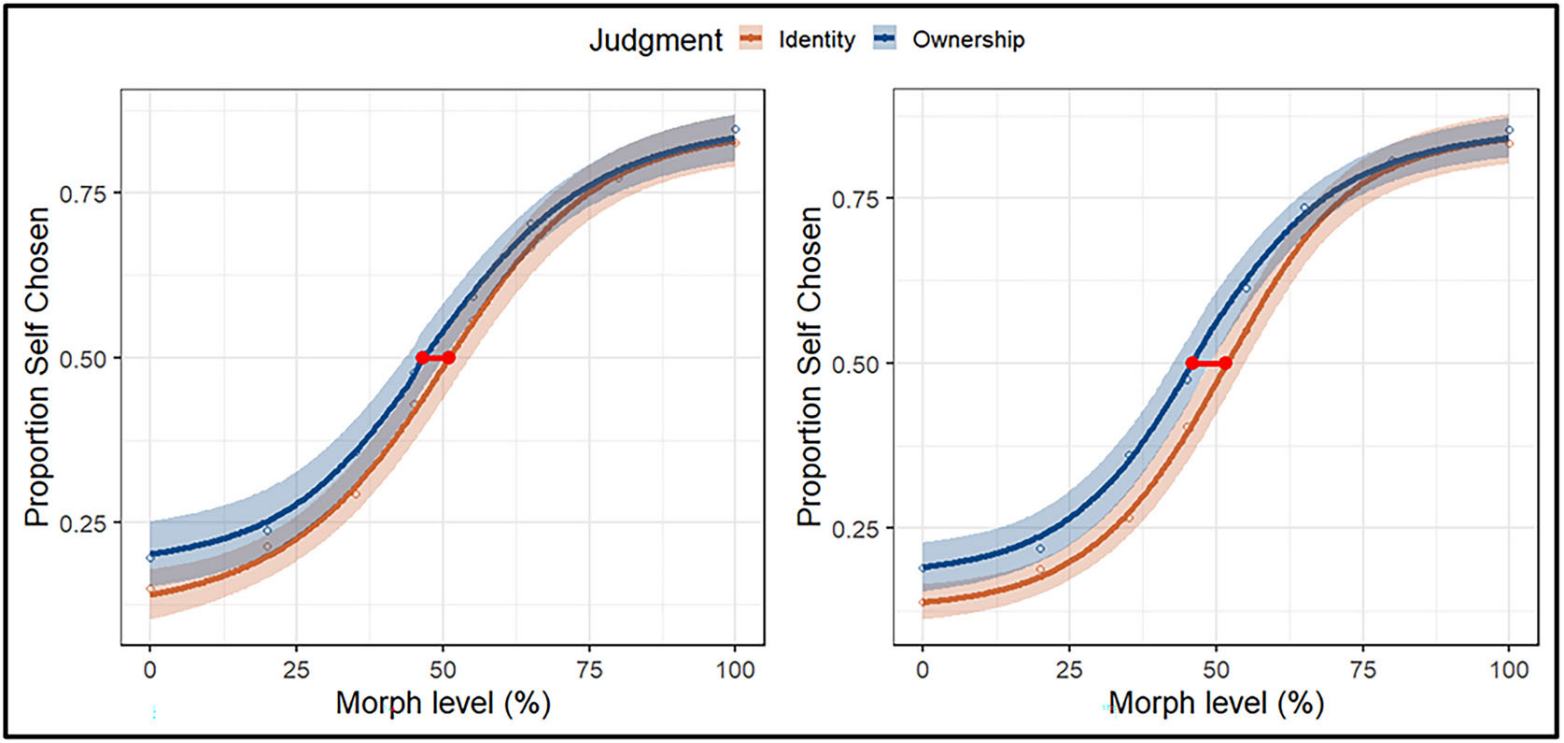
Psychometric curves as a function of Judgment (Experiment 1) for empirical (left panel) and model‐based simulated data (right panel). The y‐axis indicates the proportion of responses in which participants judged the object as self‐owned. The horizontal red line intersecting both curves illustrates the difference between judgments in the percentage of information required to reach subjective equality (i.e., a 0.5 response rate). Shaded areas represent 95% confidence intervals.

Psychometric functions were plotted with the probability of classifying an image as self‐owned on the y‐axis, scaled from 0 (the image was never classified as self‐owned) to 1 (the image was always classified as self‐owned; see also Constable et al., 2019, Truong, Roberts, & Todd, 2017). The x‐axis represents the morph continuum, ranging from 0% (e.g., an unaltered exemplar of the friend‐owned object) to a 100% (e.g., an unaltered exemplar of the self‐owned object). Intermediate values correspond to graded blends in which progressively larger proportions of self‐object pixels replaced friend‐object pixels in the images (see Fig. 2, left panel). The effect of ownership on the self‐friend distinction was quantified using the PSE, the morph level at which an image was equally likely (i.e., 50%) to be classified as owned‐by‐self or owned‐by‐friend. If self‐relevance facilitated decisional processing, then subjective equality should occur when less self‐object (vs. friend‐object) information was included in the image. Separate cumulative logistic functions were fitted for each participant and judgment (i.e., ownership vs. identity) using the *quickpsy* package (v 0.1.6; Linares & López‐Moliner, 2016). Five participants (four in the ownership‐judgment condition) were excluded from the analysis due to random responding (i.e., 50%) across morph levels. Responses faster than 200 ms were excluded from the analysis, eliminating approximately 1% of the overall number of trials.

A one‐sample *t*‐test against the objective mid‐point (50%) revealed a significant leftward shift in the PSE when ownership was probed, *t*(34) = −2.65, *p* = .012, *d* = 0.45 (*M* = 45%, *SD* = 12%). In contrast, when identity was assessed, no reliable deviation from the mid‐point was observed, *t*(38) = 1.04, *p* = .307, *d* = 0.17 (*M* = 52%, *SD* = 12%). A direct comparison using an independent samples *t*‐test confirmed a significant shift in the PSE as a function of Judgment. Specifically, subjective equality was lower in the ownership than identity condition, *t*(73) = 2.65, *p* = .010, *d* = 0.62 (*∆M* = 7%, 95% CI: 2%, 13%). These findings indicate that less diagnostic information was needed to classify self‐owned items, but only when ownership comprised the requested judgment.

### 5.2 Computational modeling

To identify the cognitive operations underpinning task performance, data were submitted to an HDDM analysis, which allows for simultaneous estimation of latent decision parameters across conditions (Wiecki et al., 2013). This approach offers benefits over conventional analyses as it decomposes observable behavior into interpretable components: drift rate (*v*), boundary separation (*a*), starting point (*z*), and nondecision time (*t*_0_). Here, we adopted the method developed by Ratcliff (2014) to generate psychometric functions based on drift rate. This approach leverages both response time and accuracy to estimate drift rate across stimulus conditions, thereby expanding the evaluable range of performance. Psychometric drift functions are particularly useful in task contexts in which stimulus quality varies parametrically along a continuum. When accuracy saturates, traditional psychometric curves flatten, thus obscuring nuanced differences in stimulus processing. In contrast, drift rates continue to vary with changes in response time, yielding a more sensitive and theoretically grounded metric of decision quality.

To probe the origins of self‐prioritization, three models were compared. Models were response coded, such that the upper threshold corresponded to self‐related responses and the lower threshold to friend‐related responses (Golubickis et al., 2018). Each model was estimated using hierarchical Bayesian inference, enabling robust parameter estimation even with modest trial counts per condition. Bayesian posterior distributions were modeled using a Markov Chain Monte Carlo (MCMC) with 10,000 samples (including 1000 burn), with outliers (5% of the trials) removed by the HDDM software (Ratcliff & Tuerlinckx, 2002; Wiecki et al., 2013).

*Model 1*.  Model 1 comprised a baseline model in which only drift rate (*v*) varied as a function of the experimental manipulations (e.g., Judgment and morph levels). Guided by a perceptual/attentional account of self‐bias (Humphreys & Sui, 2016; Sui & Humphreys, 2015), this model evaluated the possibility that self‐prioritization was underpinned by differences in the quality of evidence accumulation (i.e., a stimulus‐bias) during decision‐making. Importantly, the model assumed an unbiased starting point midway between the response boundaries (i.e., *z* = 0.50).

*Model 2*.  Extending the baseline model, Model 2 also allowed the starting point (*z*) to vary as a function of Judgment. In so doing, the model captured potential response‐selection biases—such as a prestimulus preference toward self‐related items—thus examined the possibility that self‐prioritization was underpinned by a decisional strategy (Golubickis et al., 2018).

*Model 3*.  Model 3 also allowed boundary separation (*a*) to vary across conditions. As such, the model assessed whether differences in overall response caution contributed to performance.

Model comparison was performed using the deviance information criterion (DIC), with lower values indicating better model fit. This revealed that Model 2 yielded the best fit (respective DICs: 28,148, 27,513, 27,606). For the best‐fitting model, posterior predictive checks were conducted. Specifically, we used the posterior parameter distributions from the model and simulated 10 data sets to verify that the model could reproduce key features of the accuracy data. The *quickpsy* package was used to produce the psychometric functions (see Fig. 2, left panel). Visual inspection of the simulated data confirmed the model's ability to reproduce the observed behavioral effects (see Fig. 2, right panel).

**Fig. 3:**
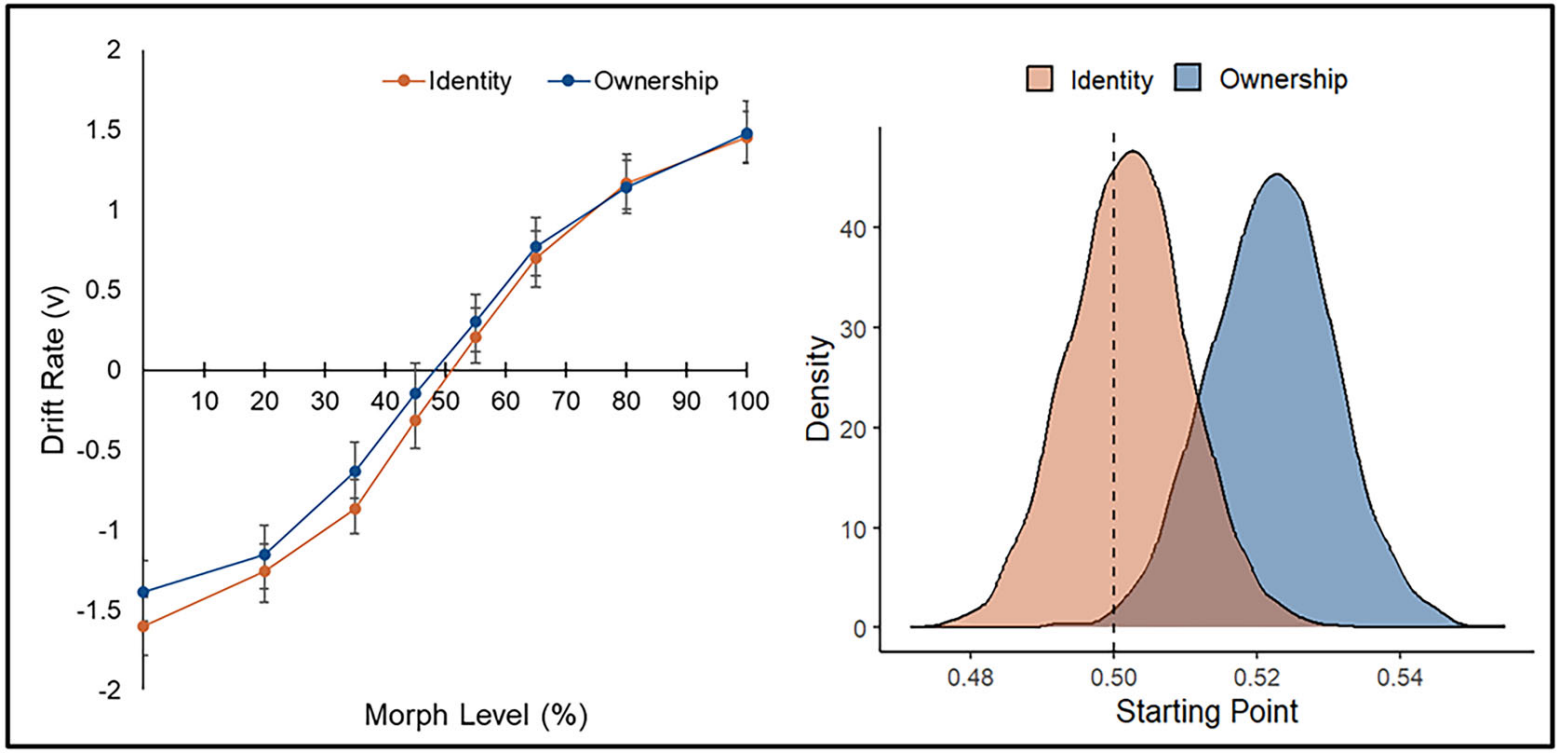
Drift‐based psychometric functions from the best‐fitting model (left panel). Error bars represent 95% HDIs. Drift rate (*v*) is plotted as a function of morph level (% self) separately for each judgment, capturing how evidence accumulation varied as a function of stimulus ambiguity. Mean posterior distributions of starting point (*z*) as a function of Judgment (right panel). The dashed line represents no bias (*z* = 0.50).

Examination of the posterior distributions revealed differences in drift rate (*v*) and starting point (*z*). Drift‐based psychometric plots—displaying drift rate as a function of morph level and Judgment—are presented for the best‐fitting model (see Fig. 3, left panel) and offer a process‐level analogue to classic psychometric functions (Ratcliff, 2014). These plots enable a granular visualization of how decision quality varied as a function of stimulus ambiguity, owner, and judgment. First, there was extremely strong evidence that drift rate varied across the morph levels (*p_Bayes_* [% self] < .001, BF_10_ > 1000), such that information uptake was faster as stimulus ambiguity decreased for both self‐owned (positive drift rate) and friend‐owned (negative drift rate) objects. Second, there was strong evidence that information uptake was faster for judgments of identity than ownership (*M*s: 0.95 vs. 0.87, *p_Bayes_* [identity > owner] = .045, BF_10_ = 21). Finally, strong evidence was observed for a difference in starting point as a function of Judgment (see Fig. 3, right panel), indicating a prestimulus preference for self‐owned responses when ownership was probed (*M*s: 0.52 vs. 0.50, *p_Bayes_* [owner > identity] = .045, BF_10_ = 21).

To establish any relationship between task performance and starting point differences, a regression analysis (i.e., HDDMRegressor) was conducted using the PSE as a continuous predictor of *z*. The analysis yielded extremely strong evidence for such a connection, with reductions in the PSE associated with increases in the starting point of evidence accumulation (*p_Bayes_* (*z* ∼ PSE) < .001, BF_10_ > 1000). This indicates that behavioral shifts in task performance were linked with the strength of participants’ preference for self‐owned responses.^1^

Collectively, the results of Experiment 1 demonstrated that personal possession influenced object classification via a self‐centric decisional strategy, but crucially only when this tactic was applicable to the decision‐making task at hand (Caughey et al., 2021; Constable et al., 2019; Falbén et al., 2019; Golubickis et al., 2018). Ownership failed to facilitate object identification, thereby confirming the goal‐dependent nature of self‐prioritization (Golubickis & Macrae, 2023). The results also indicated more efficient information uptake during identity compared to ownership judgments. This derives from differences in the difficulty of the tasks. Whereas the ownership task required stimulus recognition in combination with the attribution of ownership, the identification task necessitated only the former process (Voss, Voss, & Lerche, 2015).

In addition to task‐relevance, decisional strategies are also sensitive to the characteristics of the environment in which the decision‐maker is embedded, such that heuristics are most useful when time pressures exist, attentional capacity is compromised, or sensory inputs are impoverished (Gigerenzer, 2008; Gigerenzer & Gaissmaier, 2011). This observation has obvious implications for the adoption of a self‐centric decisional strategy during object‐ownership tasks. Notably, such a tactic should be most useful (and influential) under conditions of high compared to low decision‐making demands. In our next experiment, we explored this possibility by creating task settings in which the challenges of decision‐making varied. Specifically, participants always provided assessments of ownership (i.e., a self‐centric decisional strategy was applicable), but the demands of decision‐making were manipulated by varying the presentation duration (i.e., short vs. long) of the to‐be‐judged stimuli (Falbén et al., 2020). It was expected that self‐prioritization (i.e., shift in the PSE) would be moderated by the characteristics of the decision‐making environment, such that bias would be elevated when the task was more challenging. To identify the processes underpinning decision‐making, data were once again submitted to an HDDM analysis. Replicating Experiment 1, self‐prioritization was expected to be underpinned by a prestimulus preference for self‐owned (vs. friend‐owned) responses.

## 6 Experiment 2

### 6.1 Method

#### 6.1.1 Participants and design

Eighty participants (57 female, *M*_age_ = 22.04, *SD* = 2.95) took part in the research, all of whom had normal or corrected‐to‐normal visual acuity and received £10 (∼$13) for their assistance. Informed consent was obtained from participants prior to the commencement of the experiment, and the protocol was reviewed and approved by the Ethics Committee at the School of Psychology, University of Aberdeen, Scotland. The experiment had 2 (Presentation Duration: short or long) X 2 (Owner: self or friend) mixed design with repeated measures on the second factor. The sample size calculation was as in Experiment 1.

#### 6.1.2 Stimulus materials and procedure

Participants arrived at the laboratory individually, were greeted by a researcher, seated at a desktop computer, and informed that the experiment comprised an object‐classification task. The procedure was as in Experiment 1, but with a couple of modifications. First, all participants were instructed to report to whom the items belonged (i.e., owned‐by‐self vs. owned‐by‐friend). Second, the demands of decision‐making were manipulated by varying the presentation duration of the to‐be‐judged items, with participants randomly assigned to either the short (i.e., 50 ms) or long (i.e., 400 ms) exposure condition. Participants initially performed 16 practice trials, followed by seven blocks of 80 trials in which all stimuli appeared equally often (i.e., 10 times per block). On completion of the task, participants were debriefed, thanked, and dismissed.

## 7 Results and discussion

### 7.1 Object classification

As in Experiment 1, the psychometric functions plotted the probability of a self‐owned response as a function of morph level, ranging from 0 (never classified as self‐owned) to 1 (always classified as self‐owned). The effect of ownership on the self‐friend distinction was indexed by the PSE, the morph level at which participants were equally likely to generate self‐owned or friend‐owned responses. Separate cumulative logistic functions were fitted for each participant and presentation duration using the *quickpsy* package (v0.1.6; Linares & López‐Moliner, 2016). Twelve participants were excluded (four in the short‐duration condition) for failing to follow the instructions. Responses faster than 200 ms were excluded from the analysis, eliminating approximately 1% of the overall number of trials.

A one‐sample *t*‐test against the objective mid‐point (50%) revealed a significant leftward shift in the PSE when the presentation duration was short, indicating self‐prioritization, *t*(35) = −6.34, *p* < .001, *d* = 1.06 (*M* = 43%, *SD* = 6%). When the presentation duration was long, a similar effect was observed, *t*(31) = −1.82, *p* = .039, *d* = 0.31 (*M* = 47%, *SD* = 7%). A direct comparison of these effects confirmed that self‐prioritization was amplified when the presentation duration was short compared to long, *t*(67) = 2.33, *p* = .023, *d* = 0.58, ∆*M* = 4%, 95% CI [1%, 8%]. These findings indicate that less diagnostic information was required to classify self‐owned items when the demands of decision‐making increased.

### 7.2 Computational modeling

**Fig. 4:**
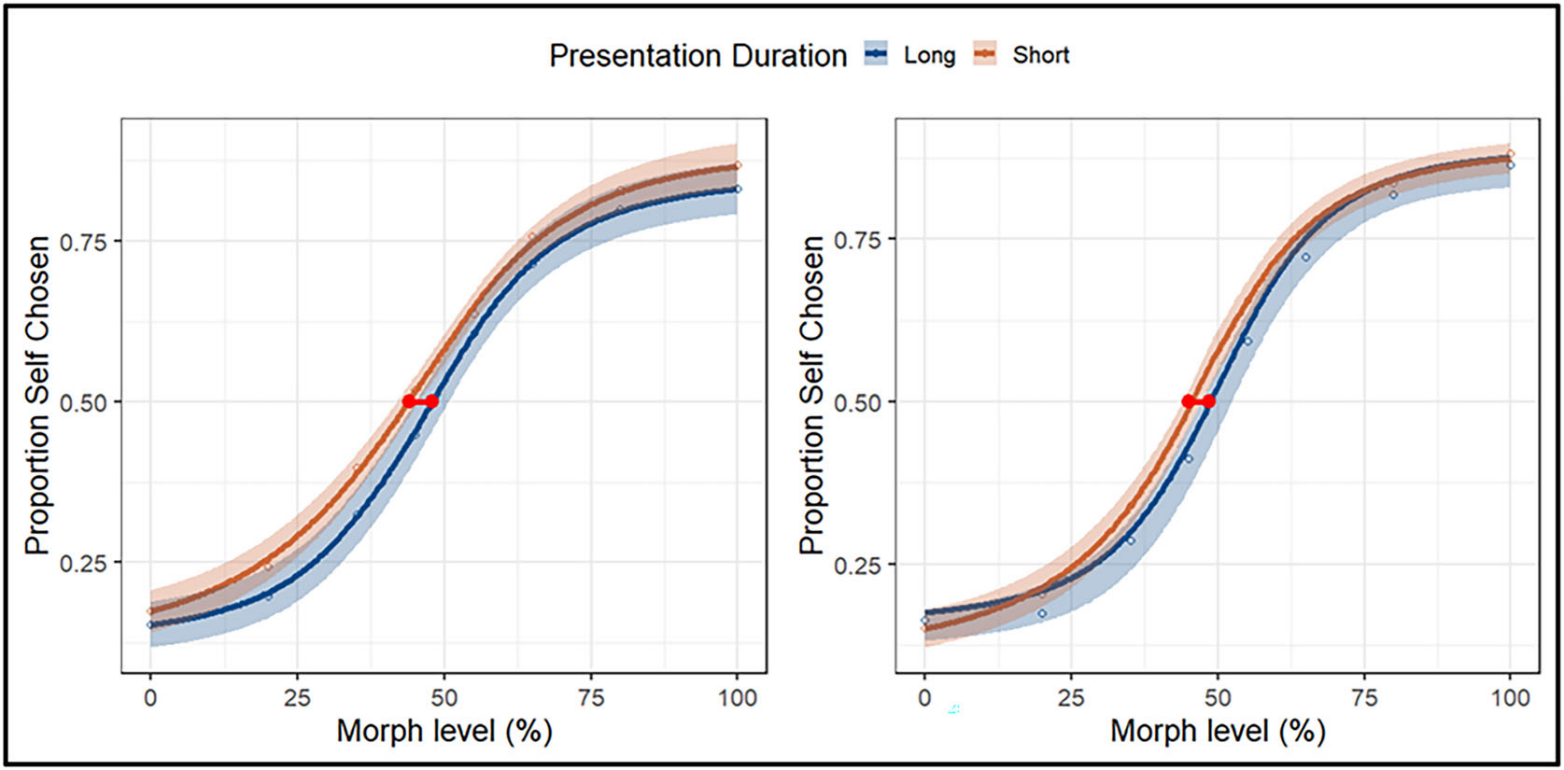
Psychometric curves as a function of Presentation Duration (Experiment 2) for empirical (left panel) and model‐based simulated data (right panel). The y‐axis indicates the proportion of responses in which participants judged the object as self‐owned. The horizontal red line intersecting both curves illustrates the difference between exposure durations in the percentage of information required to reach subjective equality (i.e., a 0.5 response rate). Shaded areas represent 95% confidence intervals.

As in Experiment 1, the data were modeled using HDDM (Wiecki et al., 2013), with parameter estimation conducted via Bayesian MCMC sampling. Three models were compared, progressively allowing drift rate (*v*), starting point (*z*), and boundary separation (*a*) to vary by condition. In this experiment, the key manipulation was Presentation Duration (short vs. long). Model comparison using the DIC again revealed that Model 2 (*v* + *z*) yielded the best fit (respective DICs: 14,842, 14,335, 14,432). Drift‐based psychometric plots were generated showing how drift rate varied as a function of morph level and Presentation Duration (see Fig. 4, left panel). Posterior predictive checks were performed using simulations from the parameter estimates of the best‐fitting model to assess its ability to reproduce the accuracy data. Psychometric functions were generated using *quickpsy* (Linares & López‐Moliner, 2016). Visual inspection of the simulated data confirmed the model's ability to reproduce the observed behavioral effects (see Fig. 4, right panel).

**Fig. 5:**
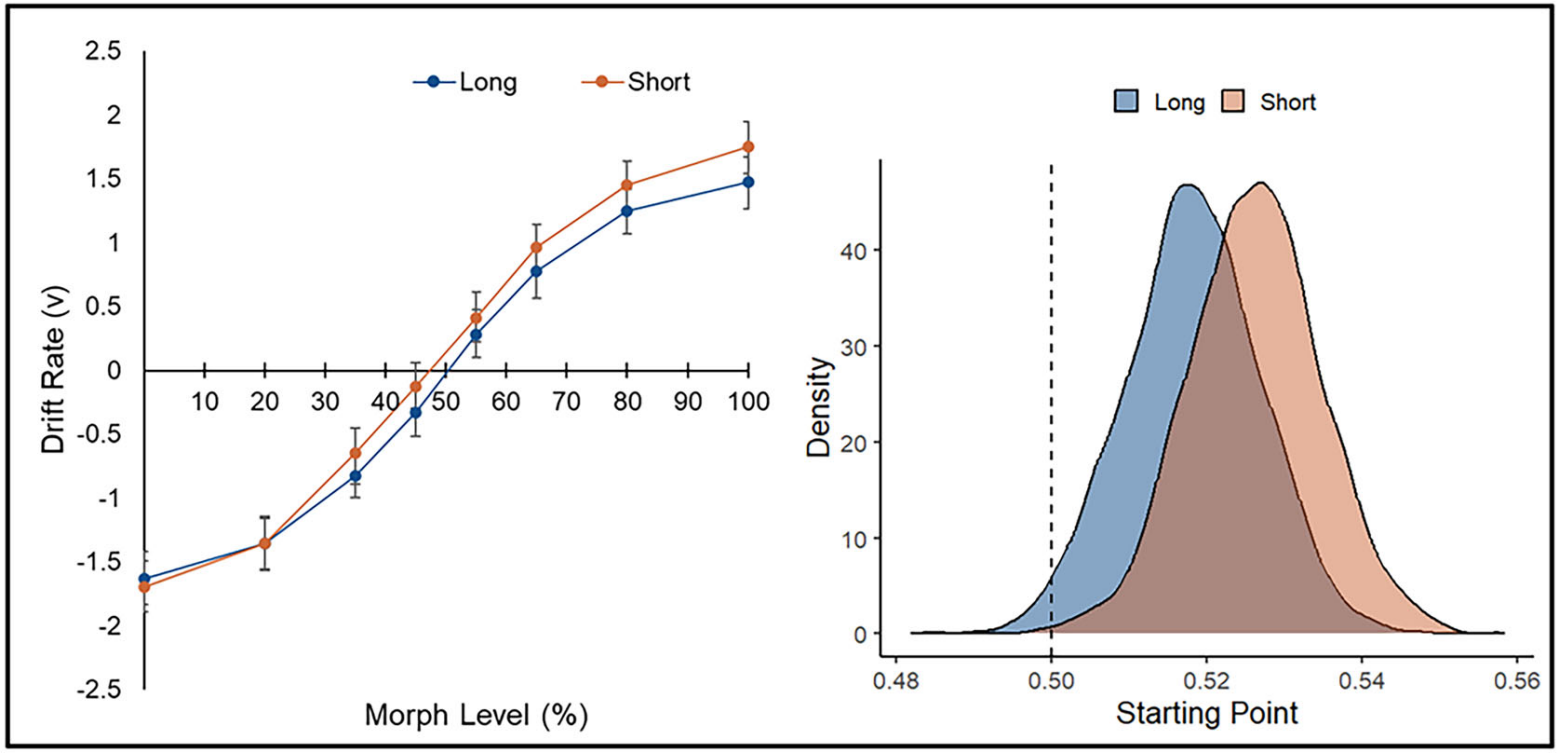
Drift‐based psychometric functions from the best‐fitting model (left panel). Error bars represent 95% HDIs. Drift rate (*v*) is plotted as a function of morph level (% self) separately for each presentation duration, capturing how evidence accumulation varied as a function of stimulus ambiguity. Mean posterior distributions of starting point (*z*) as a function of Presentation Duration (right panel). The dashed line represents no bias (*z* = 0.50).

Posterior distributions revealed differences in *v* and *z*. First, drift rate varied across the morph levels (*p*_Bayes_[% self] < .001, BF_10_ > 1000), indicating faster information uptake for both self‐owned and friend‐owned objects as stimuli decreased in ambiguity (see Fig. 5, left panel). Second, information uptake was faster under the short (vs. long) presentation duration (*M*_short_ = 1.05 vs. *M*_long_ = 0.99, *p*_Bayes_ [long < short] = .11, BF_10_ = 8) (see Fig. 5, right panel). Third, both conditions yielded a response‐selection bias (*p*_Bayes_ [short > 0.5] = .002, BF_10_ = 499; *p*_Bayes_ [long > 0.5] = .019, BF_10_ = 52). Crucially, these effects differed as a function of Presentation Duration (*M*_short_ = 0.53, *M*_long_ = 0.52, *p*_Bayes_ [short > long] = .081, BF_10_ = 11), such that the bias toward self‐owned responses was stronger when decision‐making was most challenging. To probe whether this bias tracked with differences in task performance, a regression analysis was conducted using the PSE as a continuous predictor of *z*. This yielded extremely strong evidence for such a connection, with reductions in the PSE associated with increases in the starting point of evidence accumulation (*p*_Bayes_[*z* ∼ PSE] < .001, BF_10_ > 1000). This shows that behavioral shifts in performance were linked with the strength of participants’ prior bias toward self‐owned responses.^2^

As expected, the extent to which a self‐centric decisional strategy impacted task performance was moderated by the challenges of the decision‐making environment (Gigerenzer, 2008; Gigerenzer & Gaissmaier, 2011). Specifically, the influence of personal possession (i.e., shift in the PSE) was greater when the presentation duration of to‐be‐judged objects was short (i.e., 50 ms) compared to long (i.e., 400 ms). Replicating Experiment 1, the observed SPEs were underpinned by differences in the evidential requirements of response selection (Golubickis et al., 2018), such that participants displayed a stronger prestimulus preference toward self‐owned (vs. friend‐owned) items when decision‐making was most demanding. Additionally, also as in Experiment 1, the rate of information uptake increased as the to‐be‐judged stimuli (self‐owned and friend‐owned) decreased in ambiguity. These findings further underscore the context‐dependent nature of self‐prioritization during decisional processing (Golubickis & Macrae, 2023).

## 8 General discussion

Despite widespread acknowledgment of the influence that personal possession exerts on decision‐making (i.e., self‐prioritization), the theoretical foundation and origin of this effect remains little understood. Does self‐prioritization derive from enhanced perceptual/attentional processing, the application of a strategic decisional tactic, or these factors in combination (Golubickis & Macrae, 2023; Humphreys & Sui, 2016; Sui & Humphreys, 2015, 2017)? Adopting a psychophysical approach together with computational modeling, here we showed that self‐prioritization was grounded in a response‐based decisional strategy (Gigerenzer, 2008; Gigerenzer et al., 2011). Ownership—indexed by a shift in the PSE—influenced task performance only when a self‐centric strategy was applicable (Experiment 1), an effect that was elevated under demanding processing conditions (Experiment 2). Additionally, across both experiments, self‐prioritization was underpinned by differences in the starting point of evidence accumulation, with participants exhibiting a preference for self‐owned compared to friend‐owned responses prior to the presentation of the to‐be‐judged stimuli (Falbén et al., 2020; Golubickis et al., 2018, 2019, 2021, 2023). In terms of stimulus‐based effects, the rate of information uptake increased as the images of both self‐owned and friend‐owned objects decreased in ambiguity. Collectively, these findings underscore the cognitive pathway through which ownership triggers self‐prioritization during decision‐making.

## 9 Egocentrism and decisional bias

At first glance, adoption of a self‐centric decisional strategy would appear to be counterproductive. After all, given its association with error and bias, egocentrism is surely to be avoided (Royzman et al., 2003). Echoing Tversky and Kahneman's (1974) viewpoint that decision‐making shortcuts (i.e., heuristics) engender suboptimal outcomes, work on self‐function has advanced a similar narrative. For example, when asked to report people's thoughts and feelings, self‐knowledge routinely serves as an anchor on which social judgments are based to the exclusion of other apposite person‐related information (Epley & Gilovich, 2004). As a result, people overestimate the extent to which others share their beliefs and opinions, assume others can access their private mental states, and take unwarranted credit for collaborative activities (e.g., Gilovich et al., 1998; Ross et al., 1977; Ross & Sicoly, 1979). Interestingly, however, although clearly leading decision‐making awry on occasion, it has been suggested that the benefits of egocentric strategies potentially outweigh the reported costs (Kruger, Windschitl, Burrus, Fessel, & Chambers, 2008; Moore & Small, 2007; Tamir & Thornton, 2018; Windschitl, Rose, Stalkfleet, & Smith, 2008; Zhao, et al., 2023). Specifically, when faced with uncertainty, limited attentional resources, and incomplete information, self‐centrism reduces the computational burden of social judgment, enabling goal‐relevant inferences to be generated.

Extending previous research on the topic, the current investigation provided a theoretically grounded, process‐based account of when and how self‐centrism influences decisional processing. A core feature of the adaptive toolbox is that heuristics enable people to make efficient and effective judgments under conditions of uncertainty (Gigerenzer, 2008; Gigerenzer & Gaissmaier, 2011; Gigerenzer & Goldstein, 1996). In the context of object‐ownership tasks, the “it's probably mine” heuristic serves just such a function. Uncertainty in this paradigm derives from the characteristics of the environment in which decision‐making is probed. Namely, while participants are informed which objects are owned by self and friend, no other task‐related information is typically provided. For example, it is unknown how many self‐owned and friend‐owned stimuli will be encountered (Golubickis et al., 2018). Given this ambiguity, adoption of a self‐centric strategy simplifies decision‐making, with self‐prioritization the resulting outcome. By implication, therefore, any reduction in task‐related uncertainty should impact the utility of this tactic, hence the emergence of decisional bias. Importantly, this is exactly what has been observed. Informing participants that equal numbers of self‐related and friend‐related objects will be encountered—or that friend‐related (vs. self‐related) stimuli will predominate during the task—is sufficient to eliminate self‐prioritization and trigger friend‐prioritization, respectively (Falbén et al., 2020). In other words, a self‐centric decisional strategy is adopted only when it is useful.

A key characteristic of fast and frugal heuristics is that they are computationally specifiable. Rather than furnishing a descriptive account of the process and products of decision‐making, fast and frugal heuristics are expressed as formal models, with emphasis placed on identifying the operations through which decisions are made and the task settings in which heuristics are used (Gigerenzer, 2008; Gigerenzer et al., 2011; Gigerenzer & Gaissmaier, 2011; Gigerenzer & Goldstein, 1996). Adopting this approach, here we proposed and demonstrated that the prioritization of self‐owned (vs. friend‐owned) objects during decision‐making resided in the implementation of a simple (and formal) decisional strategy, a prestimulus preference for self‐related compared to friend‐related responses (Golubickis et al., 2018, 2019). Moreover, consistent with a heuristic account of decision‐making, this self‐centric strategy was deployed only when applicable (Experiment 1) and most influential when the challenges of decisional processing were greatest (Experiment 2). As previously noted, Falbén et al. (2020) eliminated and even reversed self‐prioritization by reducing the uncertainty of the task setting in which judgments of ownership were provided (see also Svensson et al., 2022). Importantly, these effects were similarly underpinned by malleable response‐selection biases, further underscoring the flexibility and task‐specific nature of stimulus prioritization during decisional processing (Golubickis & Macrae, 2023).

## 10 The anatomy of self‐prioritization

In the current investigation—in a task context characterized by low investment/engagement (i.e., inconsequential objects comprised the stimuli of interest)—self‐centrism provided a conduit through which decisions could be made (Golubickis et al., 2018, 2019). Although beneficial, in no sense does this imply that egocentrism is a compulsory driver of object‐ownership decisions, precipitating self‐prioritization for all classes of stimuli. Indeed, the available evidence demonstrates otherwise. Take, for example, situations in which items have implications for the self‐concept (Sedikides & Strube, 1997), as is the case when people own (or are associated with) objects that vary in desirability or value (Golubickis et al., 2021; Hu et al., 2020; Jalalian et al., 2025; Lee, Martin, & Sui, 2023; Vicovaro, Dalmaso, & Bertamini, 2022). Under conditions such as these, the magnitude and persistence of self‐prioritization are tempered by the properties of the to‐be‐judged stimuli, with objects that enhance the self‐concept exerting the greatest influence on decisional processing. Thus, rather than self‐centrism wielding a permanent (and inflexible) influence on decision‐making, its deployment is tailored to the specifics of the task environment in which ownership is assessed (Gigerenzer et al., 2011; Gigerenzer & Gaissmaier, 2011; Golubickis & Macrae, 2023).

Importantly, although ownership facilitated decisional processing via a response‐selection bias, this is not the only cognitive pathway through which self‐relevance can enhance performance. In shape‐label matching tasks, self‐prioritization has been shown to be underpinned by differences in the efficiency of stimulus processing, such that evidence is extracted more rapidly from shapes that serve as proxies for self compared to other social targets (Golubickis et al., 2017, 2020; Hu et al., 2020; Sui et al., 2023; Svensson et al., 2022). What, of course, this indicates is that self‐prioritization is not a unitary phenomenon, but rather a task‐specific product of decisional processing that is underpinned by diverse cognitive processes. Of particular importance in this regard is whether self‐prioritization is probed directly, as in ownership tasks (where “self” or “other” are the required responses) or indirectly, as in shape‐label matching tasks (where “matching” or “nonmatching” are the required responses).

This basic difference in task requirements has implications for the operations that support decisional processing, hence theoretical accounts of self‐prioritization (Golubickis & Macrae, 2023; Humphreys & Sui, 2016). Whereas ownership tasks trigger self‐prioritization via variability in the demands of response selection (Golubickis et al., 2018), shape‐label matching tasks generate decisional bias through the enhanced efficiency of attentional and working‐memory operations (Svensson, Golubickis, Johnson, Falbén, & Macrae, 2023; Yin, Sui, Chiu, Chen, & Egner, 2019). In other words, a common outcome—self‐prioritization—emerges through divergent cognitive pathways. Complicating matters, however, it should be noted that a response‐based account, specifically a self‐positivity bias, has recently been advanced for facilitated performance during a modified shape‐label matching task (Verschoor, Yurtsever, Schultz, & Hommel, 2026). As people generally represent themselves more positively than others (Greenwald & Banaji, 1995), this advantages performance during matching (vs. nonmatching) trials (i.e., positive aspects of the self‐representation correspond with the positive nature of matching responses). Interestingly, this finding suggests that response‐based operations may also contribute to the generation of SPEs in stimulus‐matching tasks, at least under certain conditions.

Notwithstanding the precise origin of self‐prioritization, research employing both ownership and shape‐label matching tasks highlights a common facet of self‐function. Here, we demonstrated that, under conditions of uncertainty, people defaulted to self‐related responses to facilitate decision‐making during an object‐ownership task (Golubickis et al., 2018). Similarly, much earlier in the information‐processing stream, research has revealed that self serves as a stable anchor during shape‐label matching tasks (Lee et al., 2023; Scheller & Sui, 2022; Sui, 2016; Sui et al., 2023). Specifically, in complex and dynamic environments, self serves as an anchor guiding the perceptual and attentional operations that bind information across contexts. As Sui and colleagues have argued, acting as a central hub, self‐relevance makes it easier for people to process, remember, and act upon information (Humphreys & Sui, 2016; Sui & Humphreys, 2015, 2017). In other words, whether at the level of stimulus processing or response selection, self serves as a fundamental social‐cognitive construct that guides thinking and doing (Golubickis & Macrae, 2023; Sui & Humphreys, 2015).

## 11 Limitations and future directions

The current investigation has several limitations on the generalizability of the findings, which highlight important avenues for future inquiry. In exploring the effects of personal possession on decision‐making, only trivial objects (i.e., pencils and pens) were employed. Although in keeping with prior work on the topic (Constable et al., 2019; Falbén et al., 2019; Golubickis & Macrae, 2022; Golubickis et al., 2023; Lockwood et al., 2018), this methodological approach overlooks the pertinent matter of how personally meaningful stimuli engage the decisional operations that underpin self‐prioritization. After all, outside the laboratory, ownership extends to a wide range of consequential objects. As previously noted, items with implications for the self‐concept—such as stimuli that vary in valence or value—exert distinct effects on decision‐making (Golubickis et al., 2021; Hu et al., 2020; Jalalian et al., 2025; Lee et al., 2023; Vicovaro et al., 2022). Recently, for example, Jalalian et al. (2025) demonstrated that although high‐ and low‐value objects both trigger self‐prioritization, the benefits of ownership are more pronounced and persistent for the former (vs. latter) stimuli. Given the applicability of fast and frugal heuristics to decision‐making in naturalistic environments (Gigerenzer, 2008; Gigerenzer & Gaissmaier, 2011; Gigerenzer & Goldstein, 1996), research should, therefore, consider how basic stimulus properties (e.g., valuable vs. invaluable, highly rated vs. poorly rated) and/or self‐object relations (e.g., chosen‐by‐self vs. gifted‐to‐self) moderate the strategic emergence of self‐prioritization during decision‐making. Work of this kind is needed as it will advance understanding of when and how self‐centrism dominates decisional processing outside the laboratory for different classes of stimuli.

By testing only participants from the UK, it is possible that the current findings have inflated the extent to which an egocentric decisional strategy underpins self‐prioritization. Grounded in different socialization experiences, cultural factors are recognized to exert a potent influence on core aspects of self‐function, including decision‐making (Han & Northoff, 2008; Kitayama & Uskul, 2011; Markus & Kitayama, 1991). Whereas Western cultures characterize the self as distinct from others (i.e., independence), Asian societies emphasize self‐other interconnectedness, particularly with close family members (i.e., interdependence). An interesting upshot of these differences is that the biasing effects of self‐relevance are believed to be reduced (or even eliminated/reversed) among Asians (Zhu & Han, 2008). At least with respect to self‐prioritization, however, the available evidence does not support this viewpoint (Golubickis et al., 2019; Sui, Sun, Peng, & Humphreys, 2014). Exploring the effects of ownership across cultures, Golubickis et al. (2019) observed self‐prioritization among both Western and Southeast Asian participants, with each group exhibiting a self‐centric response‐selection bias. Of course, as a single investigation, this work does not conclusively refute the possibility that cultural forces shape self‐bias. Indeed, it would be surprising if different styles of self‐construal failed to wield such an influence, as the effectiveness of a simplifying heuristic in any environment is determined by its cultural fit (Gigerenzer, Reb, & Luan, 2022). What is, therefore, needed is research that taps how culturally shaped experiences shape the cognitive strategies through which judgments of self and others are generated in different decision‐making settings.

In the context of ownership, here we focused on when and how a self‐centric strategy impacts decision‐making (Golubickis et al., 2018, 2019). Of equivalent significance is the question of for whom this tactic facilitates decisional processing? Individuals differ markedly in egocentrism as a function of the task settings in which decision‐making is assessed (Ford, 1979), an observation with obvious implications for the emergence of self‐prioritization. Driving the current effects was the presumption that forthcoming items in an object‐classification task were more likely to belong to self than a friend (i.e., response‐selection bias—Golubickis et al., 2018). If, therefore, the strength of this assumption varies across individuals, so too should the effects of ownership on decisional processing. A useful task for future research will be to explore the extent to which individual differences in egocentrism moderate self‐prioritization. In this respect, several interesting possibilities arise. Social value orientation (SVO) refers to the degree to which people prioritize self over others in the distribution of resources (Van Lange, 1999). Crucially, individuals with a prosocial (vs. proself) orientation strive for equality and are cognizant of the needs and concerns of others (Murphy & Ackermann, 2014). By extension, one would expect SVO to influence self‐prioritization via differences in the potency of people's prior bias toward self‐owned (vs. friend‐owned) objects. Relatedly, emphasizing the nonjudgmental evaluation of present moment thinking, mindfulness has been shown to minimize self‐other differentiation, thus egocentrism (Carmody, Baer, Lykins, & Olendzki, 2009; Farb et al., 2007; Golubickis, Tan, Falbén, & Macrae, 2016, 2023; Shapiro, Carlson, Astin, & Freedman, 2006). Even without extensive meditative training, individuals are naturally more or less mindful (Burzler, Voracek, Hos, & Tran, 2019). Of interest, therefore, would be whether these differences in trait mindfulness modulate the strategic cognitive operations that underpin self‐prioritization during decision‐making.

Lastly, conceptualizing a variant of self‐prioritization as a decisional strategy rather than a stimulus bias may inform approaches to reducing self‐centric responding in situations in which it hinders performance or has undesired effects for others (or indeed self). If self‐bias was exclusively stimulus‐based, it would be uncontrollable. However, as a malleable response‐selection bias, it should be amenable to intervention and modification. By overweighing one's own perspective, egocentric biases undervalue and underutilize advice from, and the opinions of, others (Minson, Liberman, & Ross, 2011; Yaniv & Choshen‐Hillel, 2012). Similarly, individuals tend incorrectly to believe that, compared to others, they are more likely to be cooperative and helpful, leading to decisions that are neither in their own best interests nor the interests of other people (Kogut & Beyth‐Marom, 2008). To reduce instances of egocentric decision‐making, various interventions have been shown to be effective. For example, taking the perspective of others can enhance receptivity to external advice, thus improving decision‐making (Yaniv & Choshen‐Hillel, 2012). Usefully, the current findings identify a cognitive pathway through which remedial interventions may target (and attenuate) self‐centric decisional bias.

## 12 Conclusion

Despite longstanding theoretical and practical interest in the topic, quite when and how ownership influences decision‐making remains insufficiently understood (Morewedge, 2021). Integrating insights from work on self‐function and decision‐making (Gigerenzer et al., 2011; Golubickis & Macrae, 2023; Sui & Humphreys, 2015, 2017), here we showed that personal possession triggered self‐prioritization via a controllable decisional strategy. Specifically, responses to self‐owned (vs. friend‐owned) items were enhanced as participants anticipated these objects were most likely to be presented during the task, a self‐centric tactic (i.e., heuristic) that impacted performance on a sensitive psychometric measure (i.e., PSE). Although expedient in the current experimental context, it remains to be seen whether (and to what extent) this strategy would manifest in other decision‐making environments and populations. As a flexible decisional tool, it is likely to be deployed only when useful and culturally appropriate.

## Funding statement

No financial support was received for the research.

## Conflict of interest

The authors declare no conflicts of interest that are relevant to the content of this article.

## Ethics approval statement

This study was approved by the Ethics Committee at the School of Psychology, University of Aberdeen, Scotland, UK.

## Data Availability

Data and stimuli are publicly available at the Open Science Framework and can be accessed at: https://osf.io/en298/?view_only=70f017bc30bd471e98b6b270ee49f0ea.

## Notes

1 For completeness, we also fit a model in which drift rate (*v*) was regressed onto the PSE. This model showed poorer fit than the z‐regression model (DIC = 27,513 vs. 27,500).

2 For completeness, we also fit a model in which drift rate (*v*) was regressed onto the PSE. This model showed poorer fit than the z‐regression model (DIC = 14,342 vs. 14,310).
